# BMP-2 functions independently of SHH signaling and triggers cell condensation and apoptosis in regenerating axolotl limbs

**DOI:** 10.1186/1471-213X-10-15

**Published:** 2010-02-12

**Authors:** Jean-Charles Guimond, Mathieu Lévesque, Pierre-Luc Michaud, Jérémie Berdugo, Kenneth Finnson, Anie Philip, Stéphane Roy

**Affiliations:** 1 Department of Stomatology, Faculty of Dentistry, Université de Montréal, Montreal (QC) Canada; 2 Department of Biochemistry, Faculty of Medicine, Université de Montréal, Montreal (QC) Canada; 3 Department of Surgery, Faculty of Medicine, McGill University, Montreal (QC) Canada

## Abstract

**Background:**

Axolotls have the unique ability, among vertebrates, to perfectly regenerate complex body parts, such as limbs, after amputation. In addition, axolotls pattern developing and regenerating autopods from the anterior to posterior axis instead of posterior to anterior like all tetrapods studied to date. Sonic hedgehog is important in establishing this anterior-posterior axis of limbs in all tetrapods including axolotls. Interestingly, its expression is conserved (to the posterior side of limb buds and blastemas) in axolotl limbs as in other tetrapods. It has been suggested that *BMP-2 *may be the secondary mediator of sonic hedgehog, although there is mounting evidence to the contrary in mice. Since *BMP-2 *expression is on the anterior portion of developing and regenerating limbs prior to digit patterning, opposite to the expression of sonic hedgehog, we examined whether *BMP-2 *expression was dependent on sonic hedgehog signaling and whether it affects patterning of the autopod during regeneration.

**Results:**

The expression of *BMP-2 *and *SOX-9 *in developing and regenerating axolotl limbs corresponded to the first digits forming in the anterior portion of the autopods. The inhibition of sonic hedgehog signaling with cyclopamine caused hypomorphic limbs (during development and regeneration) but did not affect the expression of *BMP-2 *and *SOX-9*. Overexpression of *BMP-2 *in regenerating limbs caused a loss of digits. Overexpression of *Noggin *(*BMP *inhibitor) in regenerating limbs also resulted in a loss of digits. Histological analysis indicated that the loss due to *BMP-2 *overexpression was the result of increased cell condensation and apoptosis while the loss caused by *Noggin *was due to a decrease in cell division.

**Conclusion:**

The expression of *BMP-2 *and its target *SOX-9 *was independent of sonic hedgehog signaling in developing and regenerating limbs. Their expression correlated with chondrogenesis and the appearance of skeletal elements has described in other tetrapods. Overexpression of *BMP-2 *did not cause the formation of extra digits, which is consistent with the hypothesis that it is not the secondary signal of sonic hedgehog. However, it did cause the formation of hypomorphic limbs as a result of increased cellular condensation and apoptosis. Taken together, these results suggest that *BMP-2 *does not have a direct role in patterning regenerating limbs but may be important to trigger condensation prior to ossification and to mediate apoptosis.

## Background

Bone morphogenetic proteins (*BMP*s) are members of the transforming growth factor-β superfamily and were first discovered when ectopic cartilage and bone formation were induced by demineralized bone matrix implanted into soft tissues of animals [1,2]. However, their biological roles go far beyond osteogenesis [3,4]. *BMP*s have been extensively studied during the development of the vertebrate limb, which has long been recognized as an excellent experimental system to study the genes and signaling pathways involved in patterning complex structures.

The first stage in which *BMP*s were assessed in limb development is the establishment of the anterior-posterior limb axis. In amniotes, the posterior region of the forming limb bud is referred to as the zone of polarizing activity (ZPA) and is an organizer of the anterior-posterior patterning of developing limbs [5]. When cells from the ZPA are grafted into the anterior region of a chick limb bud, polydactyly is induced [6,7]. Previous studies have shown that sonic hedgehog (*Shh*) is expressed exclusively in the ZPA and that implanting *Shh *expressing cells, or beads of *Shh*, in the anterior limb bud is sufficient to reproduce the polydactyly induced with ZPA transplants [5,8]. On the other hand, knockout mice for *Shh *display a major deficiency in the anterior-posterior patterning of their autopods which resemble a spike like structure [9]. Thus, *Shh *appears to be a mediator for the patterning of the anterior-posterior axis of the limb [5,8,10]. Because of the restricted expression of *Shh *to the posterior margin, it remains unclear, however, whether *Shh *induces the limb polarity directly or through a secondary signal. *BMP-2 *has received much attention as a downstream target of *Shh*. Indeed, *BMP-2 *expression overlaps spatiotemporally with the expression of *Shh *in the early developing limb bud of mammalian and avian embryos [11-15] and can be induced in chick anterior wing buds by ectopic *Shh *expression [12,13]. However, overexpression of *BMP-2 *alone does not reproduce the polydactyly obtained with *Shh *overexpression in chick embryos [16,17]. Moreover, *BMP-2 *expression is maintained in the limb buds of *Shh *knockout mice [9]. In addition, the polarity and digit identity is normal in mice with *BMP-2 *deficient limbs [18]. Hence, these data show that *BMP-2 *is not essential to relay *Shh *signaling in developing limbs.

In more advanced limb developmental stages, *BMP-2*, *BMP-4 *and *BMP-7 *are all expressed in the interdigital (ID) tissue [11,15,19]. Extirpation experiments of ID tissues have shown that anterior-posterior identity is not a fixed property of digital primordia and that the ID tissues are important for digit patterning [20]. Experiments with inhibitors of *BMP *signaling in ID tissue suggested that differential levels of *BMP*s in the ID mesenchyme may be important in directing digit patterning [20]. However, a recent study [18] with conditional knockout for *BMP-2*, *BMP-4 *or *BMP-7 *showed that these *BMP*s are not essential as signals from the ID mesenchyme for specifying digit identity but that they mediate ID programmed cell death as previously reported [21,22]. In the chicken, overexpression of dominant negative type I-A or type I-B *BMP *receptors (*dnBMPR-IA *or *dnBMPR-IB*), in embryonic hind limbs, reduced ID tissue apoptosis and resulted in webbed feet [23,24]. On the contrary, application of *BMP-2 *or *BMP-7 *in the ID tissues accelerated and intensified apoptosis [25]. In ducks [19] and bats [26], *BMP*s are also expressed in the ID tissues of developing digits. However, these animals have webbed autopods and apoptosis is absent in ID tissues, suggesting expression of *BMP*s could have other roles in digit formation.

One aspect that is clearly affected by the expression of *BMP-2 *in developing limbs is skeletal development [16,17,27-29]. It was shown, in chicks, that *BMP*s are needed for chondroprogenitor cell determination and/or condensation and subsequent differentiation into chondrocytes [30]. In mice with *BMP-2/BMP-4 *deficient limbs, differentiation of condensed prechondrocytes into chondrocytes is delayed, indicating that a threshold level of *BMP*s is required to trigger chondrogenesis [18]. In these mutants, the two posterior digits fail to form as a consequence of the misexpression of the *SOX-9 *gene. *SOX-9*, a High Mobility Group (HMG)-domain transcription factor, is the earliest marker of chondrogenesis [31], and a regulator of the type II collagen gene, which is a major constituent of cartilage [29,32]. *SOX-9 *is a known target of *BMP-2 *[33] and overexpression of *BMP-2 *in developing chick limbs induces *SOX-9 *expression which then stimulates cartilage formation [29].

Among tetrapod vertebrates, urodele amphibians (i.e. axolotls) have a unique developmental pattern of their limbs. Indeed, while amniotes and anurans develop their digits almost simultaneously with a slightly accelerated onset of posterior digit formation (digit 4 being the first digit to condense in mice) [34-36], urodeles show a reversed sequence with a more progressive/successive development of anterior digits followed by posterior digits [36,37]. Although the order of digit development is different between the aforementioned animals, the end point is the same with similar digit identity in the mature limb [38]. Moreover, even though there is no clearly defined ZPA *per se *in axolotl and newt, *Shh *is expressed, as in amniotes, in the posterior mesenchyme of their developing and regenerating limb buds [39-41]. The inhibition of *Shh *signaling with cyclopamine leads to an anterior-posterior truncation of autopod elements in the developing and regenerating axolotl limbs [42,43]. In addition, overexpression of *Shh *in the anterior mesenchyme of regenerating limbs induces polydactyly [44], showing that as in amniotes, *Shh *has a morphogenetic property in patterning the anterior-posterior axis in developing and regenerating urodele limbs.

The present study addresses the role of *BMP-2 *during limb regeneration in axolotl. We report the first cloning of an axolotl full length *BMP *cDNA (*BMP-2*). We use cyclopamine, a pharmacological inhibitor of the hedgehog signaling pathway, to show that, as in mouse, *BMP-2 *is not likely to be the secondary signal of *Shh*. *BMP-2 *is expressed in the ID tissues of developing digits in virtually all tetrapod species [11,15,19,24,26,45,46] and the expression of *SOX-9 *in these digits is linked to *BMP-2 *expression [29,30,47,48]. The fact that the sequential development/regeneration of digits in axolotl limbs is the opposite of that observed in other tetrapods (anterior digits form first in axolotls instead of the posterior digits), even though *Shh *expression is expressed on the posterior side of axolotl developing/regenerating limbs, allowed us to assess whether the expression of *BMP-2 *and *SOX-9 *correlated with digits appearance (as they condense) or whether their expression correlated with *Shh *signaling in conditions where it was normal or inhibited. The effect of *BMP-2 *or *Noggin *(a general *BMP *inhibitor) overexpression on regenerating digits was also assessed.

## Results

### Cloning and analysis of *BMP-2*

The axolotl full-length cDNA encoding *BMP-2 *was cloned, sequenced and compared to the sequence of other vertebrates. Several *BMP-2 *clones were isolated from an axolotl cDNA library. The longest clone of 2228 bp [GenBank: EU339232.1] comprising an open reading frame (ORF) of 1188 bp was selected. The analysis of this ORF, using NCBI-BLAST, showed a highly conserved sequence homology of the *BMP-2 *ORF with that of other species: 83% identity to human; 83% to mouse; and 67.3% to *Xenopus laevis*. Moreover, the 3' UTR of the *BMP-2 *gene was highly conserved with that of other species (data not shown).

The translated ORF of axolotl *BMP-2 *has a predicted amino acid sequence of 395 amino acids (Additional File 1: Figure S1) that varies by less than 1% in length when compared to that of human, mouse and *Xenopus laevis*. The amino acid sequence derived from the ORF also showed a highly conserved protein homology between species: 73% with human, 72% with mouse, and 68% with *Xenopus laevis *and resulted in an expected value (E-value) of less than 1e^-164 ^when blasted in Gen Bank. Seven highly conserved cysteine residues characteristic to all members of the *TGF-β *super family [49,50] are also present (Additional File 1: Figure S1 shaded amino acids).

### *BMP-2 *and *SOX-9 *expression during limb development

Whole-mount *in situ *hybridization was performed on developing forelimbs of axolotl from developmental stages 41 to 53. The nomenclature of Nye *et al*. [51] was used to determine the staging and to label the digits of the forelimb. Digit 1 is the anterior most (corresponding to the index side in human) and digit 4 the posterior most.

**Figure 1:**
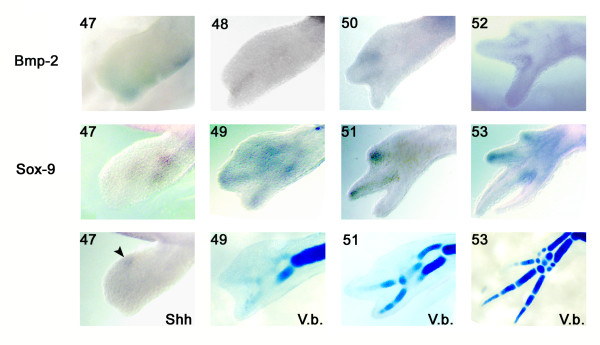
**Expression patterns (whole mount *in situ *hybridisation) for *BMP-2, SOX-9 *and *Shh *in developing limbs and cartilage staining (Victoria blue V.b.)**. All pictures show a dorsal view of the limb with the posterior side on top. Numbers in the upper left of each panel refers to the developmental stages [51].

At stage 47, the expression of *BMP-2 *was mainly detected as a narrow band that surrounded the anterior border of the limb bud and extended slightly to the posterior part of the distal end. Prior to stage 47, the expression of *BMP-2 *was expressed as a band at the edge of the limb covering both the anterior to posterior portions of the limb (data not shown) as described previously for *Xenopus laevis *[52]. *SOX-9 *was also expressed in the anterior portion of the limb bud at stage 47 while *Shh *expression is restricted to the posterior (i.e. opposite to that of *BMP-2*; Figure 1). At stage 48, the expression of *BMP-2 *had become restricted to the interdigital space between the developing digits 1 and 2 and is adjacent to the expression of *SOX-9 *in these two digits at stage 49 (Figure 1). At this stage, *SOX-9 *expression correlates with the developing cartilage elements of the wrist (carpals) and the first two digits (phalanges) (Figure 1; Victoria blue of stage 49). At stage 50, *BMP-2 *was mainly expressed between developing digits 2 and 3 and was adjacent to the expression of *SOX-9 *in these two digits at stage 51 (Figure 1). At this stage, the expression of *SOX-9 *at the tip of digit 2 correlated with the formation of skeletal elements in digit 2 (i.e. the phalanges) and the expression in digit 3 primordium was not yet associated with skeletal elements as revealed by Victoria blue staining (Figure 1; Victoria blue of stage 51). Finally, at stage 52, *BMP-2 *was expressed between developing digits 3 and 4 and was adjacent to the expression of *SOX-9 *in these two digits at stage 53 (Figure 1). At that stage, the expression of *SOX-9 *at the tip of digit 3 correlated with the formation of skeletal elements in digit 3 but the expression in digit 4 primordium was not yet associated with skeletal elements as revealed by Victoria blue staining (Figure 1; Victoria blue of stage 53). In addition to its expression between digits 3 and 4 at stage 52, *BMP-2 *expression was still detectable at the base of all 4 digits. This expression was not investigated further in the present study but has been associated with the elongation phase of digits in other species [53-55].

### Effects of blocking *Shh *signaling in developing digits

As described above, at stage 47, *BMP-2 *and *Shh *expression are on opposite sides of the developing limb bud (Figure 1; first column). It has been clearly demonstrated in mouse that *BMP-2 *is not essential for digit identity [18]. It was shown (in knockout mice for *Shh*) that *Shh *is not essential for chondrogenesis and is dispensable for *BMP-2 *expression [9]. Based on these observations, we tested, in axolotl, whether *BMP-2 *expression is independent of *Shh *signaling. The hedgehog pathway antagonist cyclopamine [56] was used to determine whether *BMP-2 *expression is independent of *Shh *signaling. The expression of *BMP-2 *and *SOX-9 *were assessed in limbs of animals treated with 2 different concentrations of cyclopamine that were shown in a previous study to prevent the formation of posterior digits [42]. In the aforementioned study, the digits that remained after cyclopamine treatment were identified as follows: when 2 digits remained after treatment, they were found to be digits 1 and 2, and when only 1 digit remained (the result of a higher concentration of cyclopamine), it was identified as digit 1 [42]. In the present study, cyclopamine treatment was initiated at developmental stage 41 which corresponds to the onset of *Shh *expression [40,51]. We confirmed that the expression of *Shh *at various limb development stages (data not shown) was has it had been reported previously by two other groups [39,40]. The group of Imokawa *et al*. also reported that *Shh *is expressed as late as the 2-3 digit stage [40] which corresponds to stage 51-52 based on Nye *et al*. [51]. The expression of *BMP-2 *and *SOX-9 *was assessed after 14 (stage 51) and 21 (stage 53) days of treatment to see whether their expression correlated with skeletogenesis even in the absence of *Shh *signaling. *BMP-2 *expression was not affected by the presence of cyclopamine prior to stage 51 of limb development (see Additional File 2: Figure S2 for the expression of *BMP-2 *at stage 45 in control and cyclopamine treated limbs). The expression of *SOX-9 *was not affected either at stage 45 in cyclopamine treated animals (see Additional File 2: Figure S2).

### 14 days

**Figure 2:**
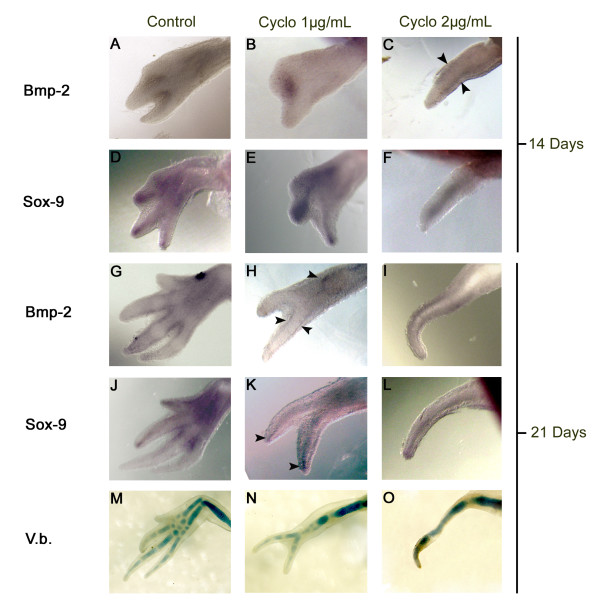
**Expression of *BMP-2 *(A-C) and *SOX-9 *(D-F) detected by whole mount *in situ *hybridisation in developing limbs treated 14 days with cyclopamine**. Expression of *BMP-2 *(G-I) and *SOX-9 *(J-L) in developing limbs treated 21 days with cyclopamine. Left column (A, D, G, J, M) shows control animals (0.4 μL EtOH/mL), middle column (B, E, H, K, N) shows the animals treated with 1 μg/mL of cyclopamine and right column (C, F, I, L, O) shows the animals treated with 2 μg/mL of cyclopamine. All pictures show a dorsal view of the limbs with posterior side on top. Arrowheads in C, H and K indicate the expression of *BMP-2 *on both sides of the regenerating digits and the expression of *SOX-9 *in the middle of the regenerating digits. The last row (M-O) shows the cartilage staining (Victoria blue) of the skeletal elements at the end of cyclopamine treatments (21 days).

After 14 days of treatment (Figure 2A-F), control animals (treated with ethanol, the carrier for cyclopamine) had reached stage 51 with digits 1 and 2 in formation (Figure 2A, D). *BMP-2 *was expressed in the interdigital space of digits 1-2 and 2-3 (Figure 2A) and *SOX-9 *was expressed at the tips of digits 1 and 2, and fully expressed in the forming digit 3 and in the carpal elements of the wrist (Figure 2D). As expected, *BMP-2 *expression was adjacent to *SOX-9 *expression in digits 1, 2 and 3. In limbs treated with 1 μg/mL of cyclopamine (Figure 2B, E), digits 1 and 2 were deformed and in animals treated with 2 μg/mL of cyclopamine (Figure 2C, F), only 1 digit was formed. The expression pattern of *BMP-2 *was adjacent to the expression of *SOX-9 *in the forming digits of cyclopamine treated animals just as it is in control animals.

### 21 days

After 21 days of treatment (Figure 2G-O), control animals had reached stage 53, and at this stage the pattern is complete. Limbs treated with 1 μg/mL of cyclopamine developed only digits 1 and 2 and limbs treated with 2 μg/mL of cyclopamine developed only digit 1. In control animals, the expression of *BMP-2 *was observed around the digits, and the expression of *SOX-9 *was in digits 3 and 4. This developmental stage corresponds to the elongation phase of digits in other species [53-55] and as previously mentioned, was not investigated further. In the animals treated with 1 μg/mL (Figure 2H, K, N) or 2 μg/mL of cyclopamine (Figure 2I, L, O) the expression of *BMP-2 *and *SOX-9 *in the digits that have formed (digits 1 and 2 for 1 μg/mL treatment and digit 1 for 2 μg/mL treatment) was adjacent, with the expression of *BMP-2 *surrounding the expression of *SOX-9 *as in the digits of control animals.

### *BMP-2 *and *SOX-9 *expression during limb regeneration

**Figure 3:**
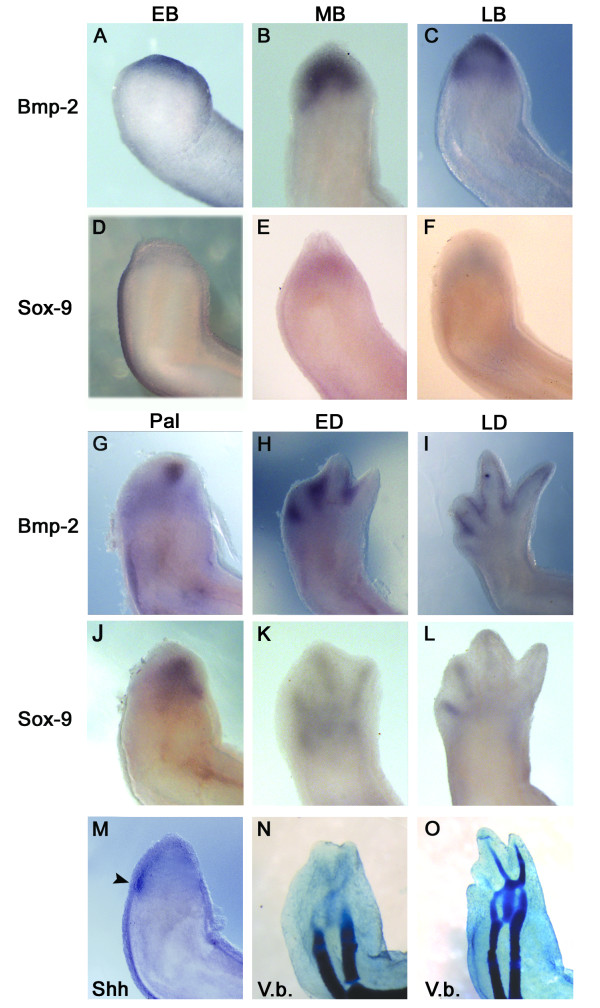
**Expression pattern, by whole mount *in situ *hybridisation, for *BMP-2 *(A-C and G-I), *SOX-9 *(D-F and J-L), *Shh *(M) and cartilage staining of skeletal elements (Victoria blue; N-O) at different stages of limb regeneration**. All pictures show a dorsal view of the regenerating limbs with posterior side on the left. EB, early bud; MB, medium bud; LB, late bud; Pal, palette; ED, early differentiation; LD, late differentiation. The arrowhead in panel M indicates the restricted expression of *Shh *in the posterior portion of the blastema.

Limb regeneration in salamander can be divided into two main phases. 1) A preparation phase that starts at amputation time and focuses mainly on the formation of the blastema; and 2) a redevelopment phase that starts approximately at the late bud stage and focuses mainly on the differentiation and repatterning of the blastema into a new limb (see reference [57] for stages description). To determine when *BMP-2 *and *SOX-9 *are expressed, whole mount *in situ *hybridisation was performed for both genes throughout limb regeneration. The onset of *BMP-2 *expression occurred at the early bud stage (EB; Figure 3A) while *SOX-9 *was not yet expressed (Figure 3D). The expression of *BMP-2 *was maintained distally in the blastema of medium bud (MB) and late bud (LB) stages (Figure 3B-C) and was adjacent to the more proximal expression of *SOX-9*; which starts at MB stage (Figure 3E-F). At the Palette (Pal) stage, *BMP-2 *(Figure 3G) and *SOX-9 *(Figure 3J) expression started to localize in the anterior region where the first two digits eventually form (as revealed by Victoria blue staining; Figure 3N). At this stage, the expression of *BMP-2 *(Figure 3G) versus that of *Shh *was on the opposite side of the autopod (Figure 3M). As in development at stage 47 (Figure 1), the opposing pattern of expression of *Shh *and *BMP-2 *just preceded the onset of digit chondrogenesis. At Pal stage, *BMP-2 *(Figure 3G) delineates the first 2 digits, and at early differentiation (ED) stage, *BMP-2 *expression also appears between digits 3 and 4 (Figure 3H). At these two stages (Pal and ED), the expression of *SOX-9 *(Figure 3J-K), was adjacent to *BMP-2 *expression (Figure 3G-H) and corresponded to the exact location of the skeletal elements. In later stages (Figure 3K-L) *SOX-9 *expression was tightly correlated to digit chondrogenesis as revealed by Victoria blue staining (Figure 3N-O).

### Effects of blocking *Shh *signaling on regenerating digits

**Figure 4:**
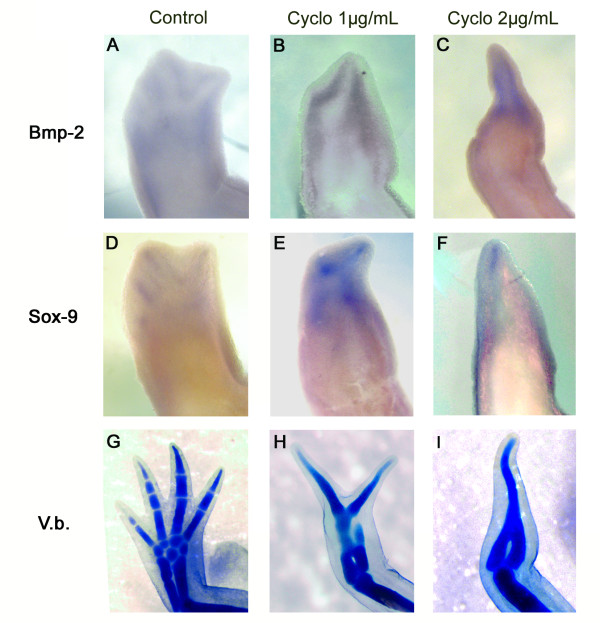
**Expression pattern, by whole mount *in situ *hybridisation, for *BMP-2 *(A-C) and *SOX-9 *(D-F) at the ED stage in regenerating limb treated with cyclopamine from the time of amputation until fixed at ED**. Left column (A, D, G) shows control animals (treated with the carrier 0.4 μL EtOH/mL), middle column (B, E, H) shows the animals treated with 1 μg/mL of cyclopamine and right column (C, F, I) shows the animals treated with 2 μg/mL of cyclopamine. All pictures show a dorsal view of the limbs with the posterior side on the left. The bottom row (G, H, I) show the skeletal staining (Victoria blue) at the end of the treatments when the control animals had completely regenerated.

As described above, at Pal stage, *BMP-2 *expression is on the opposite side of the regenerating limb bud compared to that of *Shh *(Figure 3G &3M). To determine if *Shh *signaling had an effect on *BMP-2 *expression during limb regeneration, as determined for axolotl developing limbs (Figure 2), axolotls were treated with cyclopamine. The expression of *BMP-2 *and *SOX-9 *(determined by RT-PCR at MB stage when *BMP-2*, *SOX-9 *and *Shh *are first co-expressed) was not affected after 6 or 24 hours of exposure to 1 or 2 μg/mL of cyclopamine (data not shown). The expression of *BMP-2 *at medium bud was not affected in regenerating limbs exposed to cyclopamine from the time of amputation either (see Additional File 3: Figure S3). Therefore, in order to mimic as close as possible the *Shh *knockout phenotype, axolotls were treated from the moment of limb amputation until ED (when patterning is completed). The expression of *BMP-2 *and *SOX-9 *was assessed at ED stage to determine whether their expression correlated with skeletogenesis during the redevelopment phase of regeneration as shown for development (see Figure 2). In animals treated with 1 μg/mL of cyclopamine, *BMP-2 *expression (Figure 4B) was adjacent to *SOX-9 *expression (Figure 4E). The same observation was made in the 2 μg/mL treated animals (compare Figure 4C with 4F). Under these conditions, the expression of *BMP-2 *and *SOX-9 *relative to each other was the same as observed in corresponding digits of control limbs (Figure 4A &4D). As in development, the 1 μg/mL treated animals regenerated limbs with only two digits (Figure 4H) while the 2 μg/mL treated animals regenerated limbs with only one digit (Figure 4I). Hence, the effect of cyclopamine on patterning regenerated skeletal elements as well as the expression of *BMP-2 *and *SOX-9 *were virtually identical to that observed in developing limbs treated with cyclopamine.

### Overexpression of *BMP-2 *and *Noggin *in regenerating limbs

**Figure 5:**
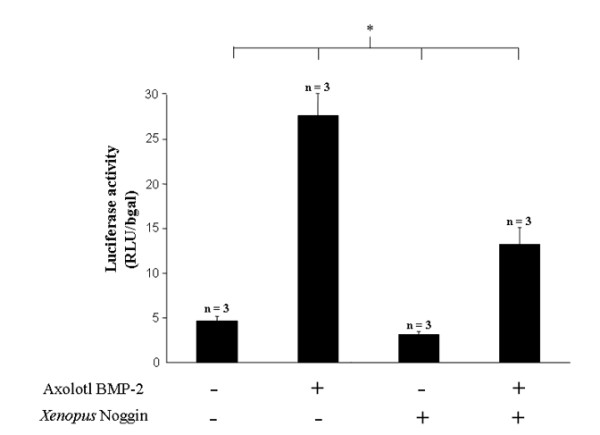
**Activation of *BMP *responsive promoter construct by ectopic expression of axolotl *BMP-2***. The graphic summarizes *BMP *responsive luciferase activity performed in the human chondrocyte cell line, C28/I2, after the transfection of *BMP-2, Noggin *or *BMP-2 *and *Noggin *expression plasmids combined. For each condition, the *BMP *responsive reporter construct and the β-galactosidase reporter construct were always co-transfected. The first bar shows the basal luciferase activity (transfected with *mRFP *without axolotl *BMP-2 *or *Xenopus laevis Noggin*) of 4.6 ± 0.6. The second bar shows a significant increase (27.7 ± 3.4) in luciferase activity when the axolotl *BMP-2 *expressing plasmid was transfected. The third bar indicates that transfection of the *Xenopus laevis Noggin *expressing plasmid decreased luciferase activity (3.1 ± 0.3) compared to basal activity. The fourth bar shows the luciferase activity from the co-transfection of the axolotl *BMP-2 *and the *Xenopus laevis Noggin *expressing plasmids. This co-expression resulted in a significant decrease in luciferase activity (13.3 ± 1.9) compared to the expression of *BMP-2 *alone. All results (mean ± sd; n = 3/condition) are expressed as relative light unit (RLU) normalised with β-galactosidase activity.

Prior to investigating the *in vivo *role of *BMP-2 *during limb regeneration, constructs expressing the monomeric red fluorescent protein (*mRFP*), *BMP-2 *and *Noggin *were tested *in vitro *to verify their efficacy (Figure 5). C28/I2 cells were cotransfected with *mRFP*, axolotl *BMP-2 *and *Xenopus laevis Noggin *along with the *BMP*-responsive BRE2-luc luciferase reporter construct [58] as well as a CMV-β-galactosidase construct as a control. Luciferase and β-galactosidase activities were determined 48 hours later and results (mean ± sd; n = 3/condition) are presented as relative light unit (RLU) normalised with β-galactosidase. The first bar shows a basal activity (*mRFP *alone) of 4.6 ± 0.6. The second bar shows that axolotl *BMP-2 *activity is significantly (p < 0.05) higher (27.7 ± 3.4) than the basal activity (an increase of 6.0 fold). In the third bar, the activity of *Xenopus laevis Noggin *(general *BMP *inhibitor) alone (3.1 ± 0.3) is significantly (p < 0.05) lower than *BMP-2 *activity and also significantly (p < 0.05) lower than basal activity. Finally, the fourth bar shows the activity (13.3 ± 1.9) resulting from *BMP-2 *in the presence of *Noggin*. The activity of this combination (*BMP-2 + Noggin*) is significantly (p < 0.05) lower than the *BMP-2 *activity alone (a decrease of 2.0 fold). Comparison between each condition (n = 3/condition) was tested using Kruskal-Wallis One Way Analysis of Variance on Ranks. Hence, these results show the plasmids expressing axolotl *BMP-2 *and *Xenopus laevis Noggin *produced functional proteins. As expected, the *BMP *responsive luciferase reporter construct was activated in the *BMP-2 *transfected cells and was antagonized in the *Noggin *transfected cells (Figure 5).

*In vivo *experiments were performed on regenerating limbs and were based on the expression pattern of *BMP-2 *&*SOX-9 *determined by whole mount *in situ *hybridisation (Figure 3). *BMP-2 *and *Noggin *containing plasmids were electroporated in the posterior-half of the blastema at the LB stages in order to obtain maximal expression of the transgene by the time the regenerates reached Pal; the stage when expression of *BMP-2 *and *SOX-9 *is localized in the anterior portion of the limb (Figure 3G &3J). Hence, the electroporation was performed to induce *BMP-2 *or *Noggin *at a time and place when no or low endogenous expression of *BMP-2 *and *SOX-9 *is detected.

**Figure 6:**
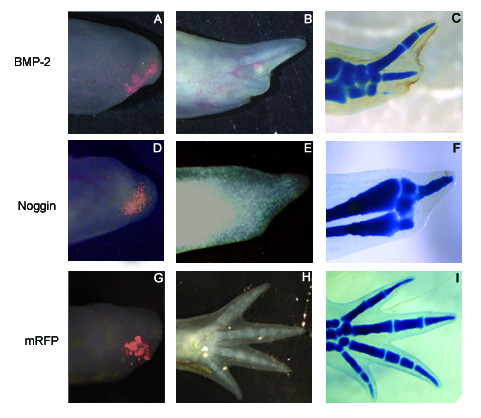
**Ectopic expression of axolotl *BMP-2 *and *Xenopus laevis Noggin *by electroporating the expression plasmids directly into regenerating limbs**. Different plasmid combinations were transfected to test the effect of *BMP-2 *and *Noggin *on the regeneration process. In order to assess the efficiency of the electroporation in vivo (first column A, D, G), the *mRFP *expressing plasmid was co-transfected with axolotl *BMP-2 *(A), *Xenopus laevis Noggin *(D) or *mRFP *alone (G; control) in the posterior half of regenerating blastema at the LB stage. The second column (B, E, H) show the resulting phenotypes once the controls (*mRFP *ectopic expression alone) were fully regenerated and the third column (C, F, I) show their skeletal elements using Victoria blue staining.

The electroporation of *BMP-2 *at the LB stage (Figure 6A) resulted in the absence of digit formation (Figure 6B-C) in the region of the electroporation (n = 6/7), while controls (electroporation of *mRFP *alone; Figure 6G) were normal (Figure 6H-I) in most cases (n = 6/7). In 1 control electroporated limb, 1 digit was missing as it sometimes happens in untreated animals (approximately 1% of the time, unpublished observations). The electroporation of *Xenopus laevis Noggin *under the same conditions (Figure 6D) resulted in a similar phenotype (Figure 6E-F; n = 4/4) to those obtained with the electroporation of *BMP-2*. When the same quantity of plasmid was injected throughout the blastema to obtain an even distribution of expression across the blastema with *BMP-2 *(n = 2/2) or *Noggin *(n = 3/4), regeneration was inhibited (data not shown). Expression of the *mRFP *construct alone throughout the blastema resulted in normally regenerating limbs (n = 4/4) therefore ruling out the electroporation itself as a cause for the inhibition of regeneration (data not shown). All electroporations performed prior to the LB stages with *BMP-2 *in the full blastema had very mild effects (n = 3/10; digits were a little shorter in the electroporated region) and in the majority of cases, had no effect (n = 7/10) (data not shown). The electroporation with *Noggin *prior to the LB stage had no effect in all cases (n = 12/12) (data not shown). In control animals the electroporation of *mRFP *prior to LB gave rise to normal limbs (n = 9/9). With every construct combination used (*BMP-2*/*mRFP *or *Noggin/mRFP *or *mRFP *alone), the fluorescence detected from the *mRFP *construct lasted a short time (5-7 days) when the electroporation was done prior to LB stage, compared to electroporation at LB stage which lasted longer (up to 15 days).

**Figure 7:**
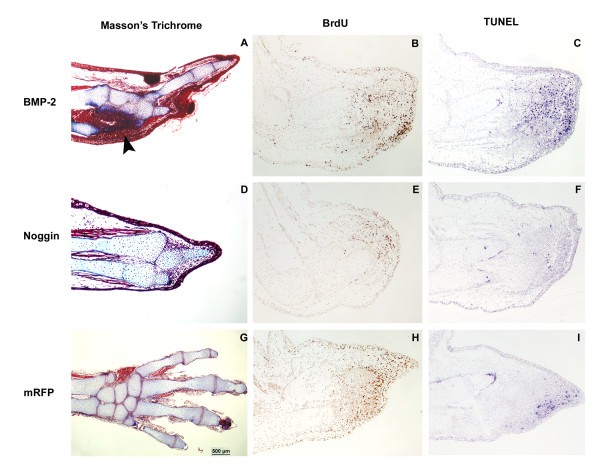
**Histological analysis, cellular proliferation and apoptosis of regenerating limbs transfected with axolotl *BMP-2*, *Xenopus laevis Noggin *and *mRFP***. Ectopic expression of axolotl *BMP-2 *(A-C), *Xenopus laevis Noggin *(D-F) and *mRFP *(G-I) by electroporating the expression plasmids directly into regenerating limbs were further analysed at the cellular level. Panels A, D and G show the resulting phenotypes at the tissue level on 10 μm sections with the Masson's trichrome staining. Clear differences can be observed between the ectopic expression of axolotl *BMP-2 *(A: condensation area pointed out by the black arrow), *Xenopus laevis Noggin *(D: no condensation visible) and the *mRFP *control (G, normal regenerate). Panels B, E and H show the level of BrdU incorporation as a measure of cell division. Overexpression of *BMP-2 *6 days after electroporation (B) did not seem to affect cell division compared to *mRFP *control (H). However, *Noggin *overexpression significantly reduced the number of BrdU positive cells 6 days after electroporation, especially on the posterior side where it was overexpressed (E). Panels C, F and I show the level of apoptosis as determined by TUNEL assay. *BMP-2 *overexpression (C) significantly increased the number of apoptotic cells during regeneration compared to *Noggin *(F), which reduced apoptosis, and *mRFP *control (I).

To investigate further the effects of *BMP-2 *miss-expression, histological analysis was performed using the Masson's trichrome staining on limbs electroporated with *BMP-2 *or *Noggin *(Figure 7A, D, G). Although the phenotypes of animals electroporated with *BMP-2 *or *Noggin *appeared similar from an exterior perspective (Figure 6), the histology results showed significant differences at the tissue level. While limbs electroporated with *BMP-2 *showed an increase in cellular density (possibly due to cellular condensation; n = 3) in the electroporated region (Figure 7A), limbs electroporated with *Noggin *(n = 3) did not show any sign of such increase in cellular density or condensation (Figure 7D). Hence, these results suggest that the similarity between the phenotypes obtained with *BMP-2 *and *Noggin *are caused by opposite effects of the ectopically expressed genes; an increase in cellular density or condensation with *BMP-2 *and a lack of cellular density or condensation with *Noggin *leading to a loss of digits.

Histological analysis provided some indication as to why *BMP-2 *and *Noggin *overexpression caused a loss of digits in regenerating limbs but it did not provide any mechanisms. Therefore, in order to help uncover the mechanism(s) as to how digit loss resulted following *BMP-2 *and *Noggin *overexpression, cell division and apoptosis were measured 6 days after electroporation of *mRFP*, *BMP-2 *and *Noggin*. Electroporation was performed at LB stage as described above and cell division was determined by 5-bromo-2-deoxyuridine (BrdU) incorporation (n = 3) and apoptosis was determined by terminal deoxynucleotidyl transferase dUTP nick end labeling (TUNEL) assay (n = 3). *BMP-2 *overexpression did not seem to cause major changes in cell division (Figure 7B) compared to the controls electroporated with *mRFP *(Figure 7H) which regenerated normally (Figure 7G). Although *BMP-2 *overexpression did not cause major changes in cell division, it did cause a marked increase in the number of apoptotic cells (Figure 7C) when compared to control electroporated with *mRFP *(Figure 7I). On the opposite, *Noggin *overexpression resulted in a marked reduction in cell division (Figure 7E) and the near absence of apoptotic cells (Figure 7F) compared to *BMP-2 *overexpression and the control electroporated with *mRFP*.

## Discussion

Our results show that the expression of *BMP-2 *and *SOX-9 *in developing axolotl digits is very similar to the expression observed in other non amphibian species (e.g. mouse & chicken) [11,15,19,24,26,29,30,45-48]. The expression pattern of these two genes is also reproduced during digit formation in regenerating axolotl limbs. Since *SOX-9 *is the earliest marker of chondrogenesis and a known target of *BMP-2*, these results suggest an activation of *SOX-9 *expression (in cells that will form the digit bones) by *BMP-2 *expressing cells (in the ID tissue).

As described by previous studies [42,43], the number of digits that form in cyclopamine treated animals (during development and regeneration) is dose-dependent. In the digits that do form in cyclopamine treated animals, the expression of *BMP-2 *and *SOX-9 *as well as chondrogenesis proceed normally. These results suggest that in axolotl just as it was observed in mice [9], *BMP-2 *is not likely to be a secondary signal for *Shh *signaling. This is also supported by the fact that when digits begin to condense in developing and regenerating limbs, *BMP-2 *and *Shh *expression are on opposite sides of the limbs (along the anterior-posterior axis) and do not overlap. Hence, these results suggest that, as in mouse, *BMP-2 *can trigger cell condensation independently of *Shh *signaling.

Our results strongly suggest that the inhibition of digit regeneration, in the limbs in which *BMP-2 *was overexpressed, is the result of increased cell condensation and apoptosis. This is also supported by a previous study showing an inhibition of chick limb development in the presence of ectopic *BMP-2 *[59]. The *BMP-2 *overexpression experiments and the complementarities of expression of *BMP-2 *and *SOX-9 *(in developing and regenerating limbs), support our hypothesis that *BMP-2 *triggers the condensation of adjacent cells destined to become chondrocytes (e.g. in the digits). Taken together, theses results suggest that the role of *BMP-2 *during limb regeneration is to trigger cell condensation and may be to control, to some extent, apoptosis. The data obtained with *Noggin *overexpression also provides further insight on the potential role of *BMP *signaling in the regeneration process. *Noggin *is an inhibitor of *BMP*s (general inhibitor of all *BMP*s) and therefore the results obtained could potentially affect *BMP*s other than *BMP-2*. However, these results indicate that *BMP *signaling is essential for cellular division and apoptosis during the regeneration process, since both processes are inhibited when *Noggin *is overexpressed.

The fact that the effects of *BMP-2 *and *Noggin *were observed when overexpressed at the LB stage suggest that *BMP-2 *regulates cellular activities during the redevelopment phase of regeneration that starts approximately at the LB stage or later (see [60,61] for a more complete description of the different phases of regeneration which are often referred to as: preparation phase which includes the initial stages leading to blastema formation; and redevelopment phase which includes the stages that give rise to the differentiated tissues and the patterning of the regenerated limb). However given that overexpression of *BMP-2 *and *Noggin *were performed using electroporation of plasmids, technical limitations do not allow us to draw any conclusion regarding the role of *BMP-2 *during the preparation phase of regeneration. Given the high level of cell proliferation and tissue reorganisation during the stages prior to the LB stage, it is possible that the electroporated plasmids could have been degraded before affecting the regeneration process or that cells compensated somehow to overcome the ectopic *BMP *signaling. This is supported by the rapid rate of fading of red fluorescence resulting from the reporter *mRFP *plasmid, when electroporations were done prior to LB stage. In addition, the fact that *Noggin *did not affect regeneration when overexpressed prior to LB stage is indicative that *BMP *signaling may not be essential for cellular proliferation during EB and MB stages.

## Conclusion

This work represents the first study on the role of *BMP*s during the process of limb regeneration in urodele amphibians. Our data demonstrate that the role of *BMP-2 *is conserved in limb regeneration as in limb development and that its function is related to skeletogenesis, cellular proliferation and apoptosis but not pattern formation, as previously demonstrated by the group of Tabin in mice [18]. The ectopic expression of *BMP-2 *led to the condensation of cells in regenerating axolotl limbs. The expression of *BMP-2 *and *SOX-9 *in addition to our functional analysis data from the ectopic expression of both *BMP-2 *and *Noggin *clearly indicate that *BMP *signaling is essential for the process of limb regeneration especially during the redevelopment phase where re-differentiation of all the different cell types and pattern formation occur.

## Methods

### Animal maintenance and surgery

Axolotl embryos and larvae (3.0 cm to 8.5 cm from snout to tip of the tail) were purchased from the *Ambystoma *Genetic Stock Center (Lexington, KY, USA). Animals were kept in 20% Holtfreter's solution at a temperature varying from 19-22°C with a photoperiod cycle of 12 hours of light and 12 hours of darkness. Embryos were used for limb development experiments and larvae for limb regeneration experiments. For all manipulations performed, axolotls were anaesthetized in 0.1% MS222 (ethyl 3-aminobenzoate methanesulfonate salt, Sigma-Aldrich, MO, USA) in Holtfreter's solution at pH 7.0. Axolotls were allowed to recover from anaesthesia in 20% Holtfreter's or were euthanized/fixed for subsequent experiments. All animal care and experiments were done in agreement with the Université de Montréal animal care committee's guidelines.

### Cloning of axolotl *BMP-2 *full length cDNA

Partial axolotl *BMP-2 *cDNA of 239 bp was obtained from axolotl larvae total RNA by RT-PCR. The 239 bp cDNA was amplified with primers (see Additional File 4: Figure S4) designed from a partial cDNA sequence of *BMP-2*/*4 *like directly submitted to Genbank by Dalglish, G. *et al*. (Accession number: AY326272). The full length *BMP-2 *cDNA was subsequently obtained by screening an axolotl cDNA library (Stratagene, CA, USA), following the manufacturer's instructions and using the 239 bp fragment as a probe radioactively labeled with ^32^P-dCTP (Perkin Elmer, MA, USA).

### Whole-mount *in situ *hybridization

Whole-mount *in situ *hybridization was performed as described in Gardiner *et al*. [62] with a few modifications: The axolotls were anaesthetized and euthanized/fixed in 1× MEMFA (10× MEM salt (1 M MOPS pH 7.4, 20 mM EGTA, 10 mM MgSO_4_), 3,7% formaldehyde in DEPC treated H_2_O) then transferred to 100% methanol and stored at -20°C until needed. Fragments of 449 bp and 381 bp (for *BMP-2 *probes), 458 bp and 455 bp (for *SOX-9 *probes) and 371 bp (for *Shh *probes) were generated by RT-PCR (see Additional File 4: Figure S4 for primers used) and cloned separately into the pCRII-TOPO cloning vector (Invitrogen, CA, USA). These plasmids were then linearized using the appropriate restriction enzymes and used as template for generating antisense RNA probes. Digoxigenin (DIG) labelled antisense RNA probes were generated using T7 or SP6 RNA polymerase (Invitrogen, CA, USA) and DIG RNA labelling mix (Roche Diagnostics, QC., Canada). Two different probes for *BMP-2 *were synthesised. The 3' UTR of the *BMP-2 *gene is highly conserved throughout evolution and it was utilized to make a highly specific probe for *BMP-2*. Thus, one probe of 381 bp was designed to overlap part of the 3' UTR and the ORF of *BMP-2*. A second probe of 449 bp was designed in the region overlapping the 5' UTR and the ORF of the gene which confirmed the expression observed with the 381 bp probe (data not shown). The expression pattern of *SOX-9 *was also determined using two different probes with identical results obtained for both probes (data obtained using the 455 bp probe are not shown). The expression of *Shh *was assessed with only one probe since the results were identical to the results published by two different groups [39-41]. For tissue permeabilization of developing embryos, the limbs were incubated with 20 μg/mL proteinase K for 15 min on ice and then at 37°C for 5 min. For permeabilization of regenerating tissues, limbs were incubated with 30 μg/mL proteinase K for 1 h on ice and then at 37°C for 45 min. Probes were hybridized 24 h for embryos and 72 h for regenerating animals. Prehybridization and hybridization temperatures were done at: 55°C for the *BMP-2 *449 bp probe and 60°C for all the other probes. Finally, BM purple (Roche Diagnostics, QC., Canada) was used as the alkaline phosphatase substrate for the colorimetric reaction. Each time point presented were performed at least 3 times.

### Whole mount skeletal staining of limbs

Cartilage was stained using the Victoria Blue method [63]. Limbs were fixed in Bouin's fixative solution for 24 h before being rinsed several times with 70% ethanol. Specimens were then rinsed in 3.5% NH_4_OH for 2 days and subsequently treated with Acid alcohol for 2 h. Specimens were stained with 1% Victoria Blue for 2 h and then rinsed with 70% ethanol. Limbs were dehydrated using a gradient ethanol series of 95% and 100%, then cleared and stored in methyl salicylate.

### Histology

Electroporated limbs were fixed overnight in Bouin's fixative solution and then rinsed thoroughly with 70% alcohol. Limbs were embedded in paraffin and cut to 10 μm sections. Slides were deparaffinised through 3 baths of toluene for 5 minutes each. Slides were then rehydrated in a graded series of 100%, 90%, 70% and 50% ethanol and then distilled water for 5 minutes each. Masson's trichrome staining method was used to stain cell cytoplasm in red, nuclei in black and collagen in blue [64].

### Cyclopamine treatments

Cyclopamine powder (Cedarlane, ON., Canada) was dissolved in 100% ethanol at a concentration of 5 mg/mL and stored at -20°C. Cyclopamine was administered by adding it directly to the Holtfreter's solution to the desired concentrations. For each conditions presented in this study, 10 animals were treated with cyclopamine or ethanol (control). For experiments on developing limbs, treatments began at the developmental stage 41 according to Nye *et al*. [51] and animals were fixed after 14 and 21 days of treatment (corresponding approximately to stage 51 and 53 in control animals). For regenerating animals, treatments began at the time of limb amputation and animals were fixed when the controls reached the palette stage (see [57] for stages description) and at completion of regeneration. For limb development as well as limb regenerating experiments, control animals were treated with the same volume of 100% ethanol that was used to dissolve the cyclopamine. Controls were done for both volumes of ethanol (0.2 μL/mL and 0.4 μL/mL) used for the 1 and 2 μg/mL cyclopamine treatment. There was no difference in limb development between these two control conditions (data not shown) and therefore only those corresponding to 2 μg/mL cyclopamine treatment are presented. When the control animals with ethanol were compared with animals bathing in ethanol free solution, there was no difference in limb patterning and gene expression. All solutions were changed daily. Animals were euthanized at the end of the experiment and fixed.

### Luciferase assays

The immortalized human chondrocyte cell line, C28/I2, has been described previously [65]. It was developed from chondrocytes isolated from juvenile human costal cartilage and immortalized by retroviral infection of the SV40 large T antigen [65]. C28/I2 cells were cultured in D-MEM/F12 containing 10% FBS with antibiotics (penstrep, fungizone) and 2 mM L-glutamine.

Transfection was performed using Lipofectamine 2000 (Invitrogen, Carlsbad, CA). BRE_2_-luc was described previously [58]. C28/I2 cells were seeded at a density of 7.5 × 10^4 ^cells in a 12-well plate. The next day, cells were transfected with the following constructs and concentrations: axolotl *BMP-2 *= 0 or 0.4 μg, *Xenopus laevis Noggin *= 0 or 0.4 μg, BRE_2_-luc = 0.35 μg and pCMV-β gal = 0.35 μg. The total DNA concentration was brought up to 1.5 μg using pTYFP (expression vector for *mRFP*) as filler DNA. Cell lysates were prepared 48 hours later and analyzed for luciferase and β-galactosidase activity as described previously [66]. Data are expressed as a mean ± standard deviation luciferase activity normalized to β-galactosidase activity. Comparison among each condition (n = 3/condition) was tested using Kruskal-Wallis One Way Analysis of Variance on Ranks.

### Injections and electroporation of constructs

For injections/electroporation of constructs expressing axolotl *BMP-2 *or *Xenopus laevis Noggin*, blastemas were injected with 1-1.5 μL of circular DNA solution through a glass needle with a 0.2-0.4 μm diameter tip, using a cell microinjector PM-1000 (Micro Data Instrument, Inc., NJ, USA). Immediately following injections, constructs were electroporated in blastemas. Thin gold plated rectangular electrodes (3 mm × 5 mm) were placed under (anode) and over (cathode) the blastema. 5 trains (1 train/second) of 20 square wave pulses (200 Hz for 100 ms), at 10 Volts peak to peak, were applied through the electrodes using a S8 stimulator (Grass instruments, MA, USA; Additional File 5: Figure S5). Vectors containing axolotl *BMP-2 *(5 μg/μL) or *Xenopus laevis Noggin *(5 μg/μL) were always co-injected with a vector containing the *mRFP *(2.8 μg/μL) to monitor the spread of the injection over time and space. Control animals were injected with 7.8 μg/μL of *mRFP *(the vector containing *BMP-2 *or *Noggin *was replaced by the same amount of *mRFP*). *BMP-2*, *Noggin *and *mRFP *expressions were all under the control of the constitutive CMV promoter in the pcDNA1/AMP expression vector (Invitrogen, CA).

### BrdU incorporation assay

Regenerating axolotls were injected intra-peritoneally, 6 days after electroporation of plasmids (as described above); with 10 μL of BrdU stock solution using a ratio of 1-2 mL/100 g of body weight according to the manufacturer's instructions (GE Healthcare, # RPN201). Animals were fixed 12 hours after injection in 4% paraformaldehyde in 0.7× PBS for 24 hours at 4°C. Samples were paraffin embedded, deparaffinized and rehydrated as for histology. For immuno-histochemistry, slides were washed 4 × 15 minutes in PBST (1× PBS with 0,1% Tween 20). They were incubated with 0.8% Pepsin in 0,2N HCl for 10 minutes at 37°C to promote denaturation of DNA. Slides were then washed 3 × 10 minutes in PBST, and incubated in a blocking solution (2% bovine serum albumin, 1% DMSO, 10% sheep serum and 0,1% Triton X-100) at room temperature for one hour. Slides were then incubated overnight at 4°C with an anti-BrdU mouse monoclonal antibody (BrdU Ab-3, Labvision/Neomarkers, Fremont, CA.) diluted 1:50 in blocking solution. PBST washes (4 × 15 minutes) were done before incubating with an anti-mouse secondary antibody coupled to horseradish peroxidase, dilution 1:250, for 2 hours at room temperature (GE Healthcare). Slides were washed again 4 × 15 minutes in PBST before being incubated in DAB (Zymed, Invitrogen CA) for signal detection. Slides were counterstained with methyl green for 6 minutes (Dako, Mississauga, ON) before being serially dehydrated 2 × 2 minutes each in 90% EtOH, 100% EtOH and 100% Xylene. Slides were mounted with Permount (Fisher scientific, Ottawa, ON). Immunohistochemical detection for BrdU incorporation was done on three different samples for each condition tested.

### TUNEL assay

TUNEL assay was adapted from Vlaskalin *et al*. [67] with minor modifications. Tissue sections were deparaffinised and rehydrated as for histology. In a humidified chamber, sections were incubated for 20 minutes in TBS (Tris-buffered saline: 100 mM Tris-HCL pH 7.5 + 150 mM NaCl), containing 20 μg/ml proteinase K at RT and rinsed 3 × 1 minute in TBS. Slides were rinsed in TBS, equilibrated in 1× TdT buffer for 5 minutes and incubated in TdT buffer containing 75 U/ml TdT and 2 μM digoxygenin-dUTP for 1 h at 37°C. End labelling was stopped by transferring the sections into 1× TBS/1 mM EDTA for 10 minutes at RT, followed by 2 × 2 minutes rinses in TBS. Sections were equilibrated for 5 minutes in TBS and incubated in blocking solution [2% sheep serum in TBS] for 1 h at RT. Tissues were incubated overnight at 4°C in blocking solution containing 1:1500 dilution of anti-DIG-AP. The following day, sections were rinsed in PBS and equilibrated 5 minutes in AP buffer. The chromogenic reaction was carried out in AP buffer containing 100 mg/ml NBT and 50 mg/ml BCIP. The reaction was monitored for up to 15 minutes and stopped by several washes in 1× PBS.

### Image treatments

Pictures of whole mounts, Victoria blue staining, and mRFP fluorescence were taken by a digital infinity 2 camera (Lumenera corporation, ON., Canada) through a Leica MZ-16F binocular (Leica Microsystems Ltd., Switzerland). Images of fluorescence in regenerating limbs were merged with white light images using Photoshop 7.0.1 (Adobe Systems Inc., CA, USA).

## Authors' contributions

J-CG carried out the majority of manipulations, participated in the design of the study and drafted the manuscript. ML performed substantial work in the histology experiments and helped in reviewing the manuscript. P-LM cloned the axolotl *BMP-2 *cDNA. JB managed the cyclopamine experiments on developing axolotls. KF performed the Luciferase assays experiments and helped in reviewing the manuscript. AP supervised the Luciferase experiments and helped in reviewing the manuscript. SR participated in the design of the present study, supervised all the experimentations and did all the major corrections of the final manuscript. All authors read and approved the final manuscript.

## Supplementary Material

Additional file 1 **Figure S1: Sequence analysis of axolotl *BMP-2***. Sequence analysis and alignment of the axolotl *BMP-2 *protein. Alignment of the predicted axolotl *BMP-2 *protein with human, mouse and *Xenopus*. Seven cysteine residues that are highly conserved and characteristic to all members of the *Tgf-β *superfamily are shaded in red. Click here for file

Additional file 2 **Figure S2: Expression of *BMP-2 *and *SOX-9 *at stage 45 of limb development**. Expression of *BMP-2 *and *SOX-9 *at stage 45 of limb development in control and cyclopamine treated (2 μg/mL) axolotls. *Shh *has been shown to be expressed at this stage of axolotl limb development. Our results indicate that the expression of *BMP-2 *and *SOX-9 *during limb development is not dependent on *Shh *signaling as they are not affected by cyclopamine treatment. Panels A and B show the expression of *BMP-2 *in control (A) and cyclopamine treated (B) animals. Panels C and D show the expression of *SOX-9 *in control (C) and cyclopamine treated (D) animals. Click here for file

Additional file 3 **Figure S3: Expression of *BMP-2 *and *SOX-9 *at MB with cyclopamine**. Expression of *BMP-2 *and *SOX-9 *at MB stage of limb regeneration in control and cyclopamine treated (2 μg/mL) axolotls. *Shh *has been shown to be expressed at this stage of axolotl limb regeneration. Our results indicate that the expression of *BMP-2 *during limb regeneration is not dependent on *Shh *signaling as it is not affected by cyclopamine treatment. Panels A and B show the expression of *BMP-2 *in control (A) and cyclopamine treated (B) animals. Panels C and D show the expression of *SOX-9 *in control (C) and cyclopamine treated (D) animals. Click here for file

Additional file 4 **Figure S4: Primers**. Primers used for PCR amplification of the different probes used for whole mount in situ hybridization and cDNA library screening. Click here for file

Additional file 5 **Figure S5: *In vivo *electroporation**. Schematic representation of the electric pulses used for electroporating the plasmids in regenerating tissues. 5 trains of pulses were applied for the *in vivo *electroporation of expression constructs. For each electroporation, a train of 20 square waves (10 V peak to peak) in 100 ms (200 Hz) is applied every second (1 Hz) over five seconds (5 trains in total). Click here for file
