# Supplementary figures and images for: BMP-2 functions independently of SHH signaling and triggers cell condensation and apoptosis in regenerating axolotl limbs
Source: BMC Dev Biol. 2010 Feb 12;10:15. doi: 10.1186/1471-213X-10-15 (PMC2829471; doi:10.1186/1471-213X-10-15)

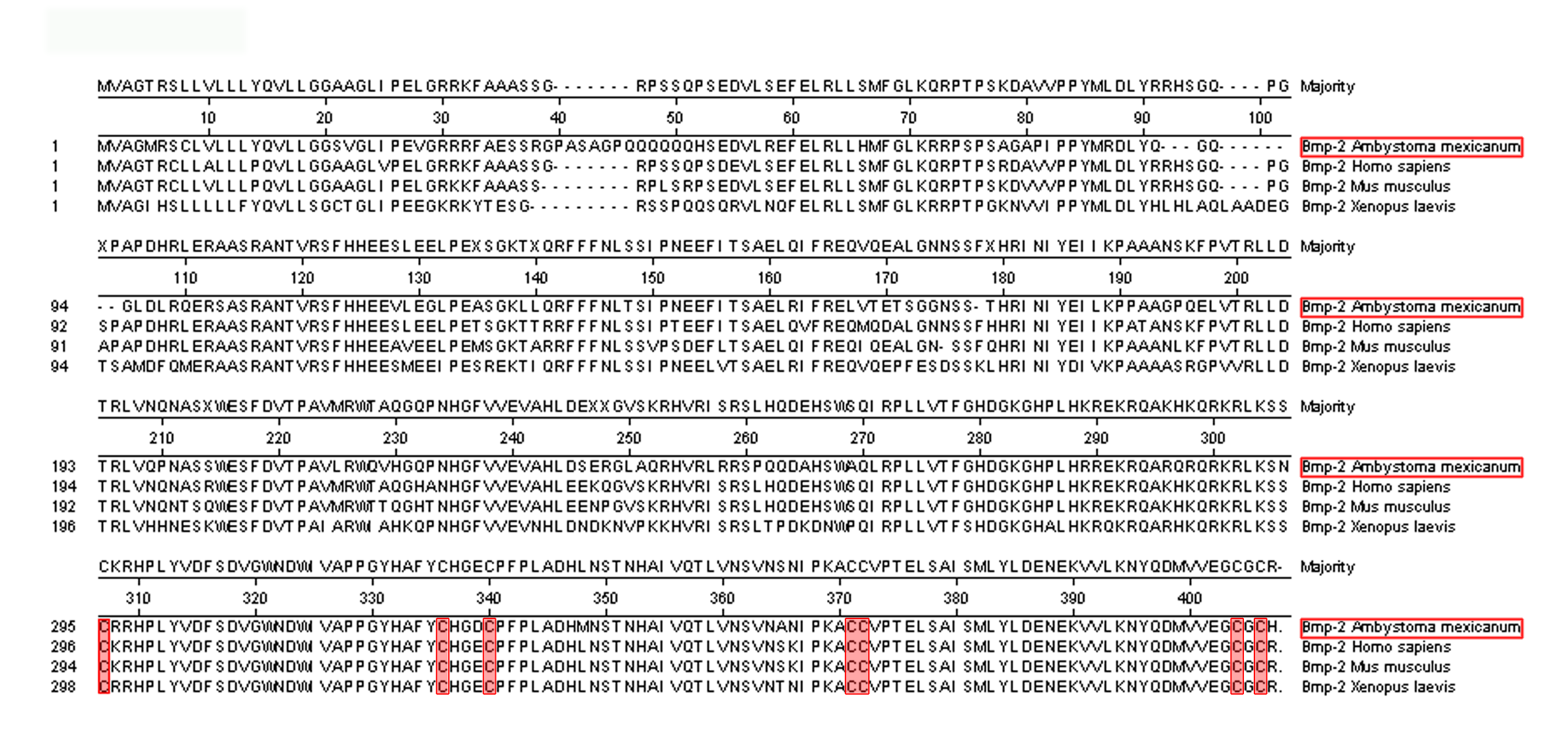

Supplement: Additional file 1 — Figure S1: Sequence analysis of axolotl BMP-2. Sequence analysis and alignment of the axolotl BMP-2 protein. Alignment of the predicted axolotl BMP-2 protein with human, mouse and Xenopus. Seven cysteine residues that are highly conserved and characteristic to all members of the Tgf-β superfamily are shaded in red. [file 1471-213X-10-15-S1.TIFF]

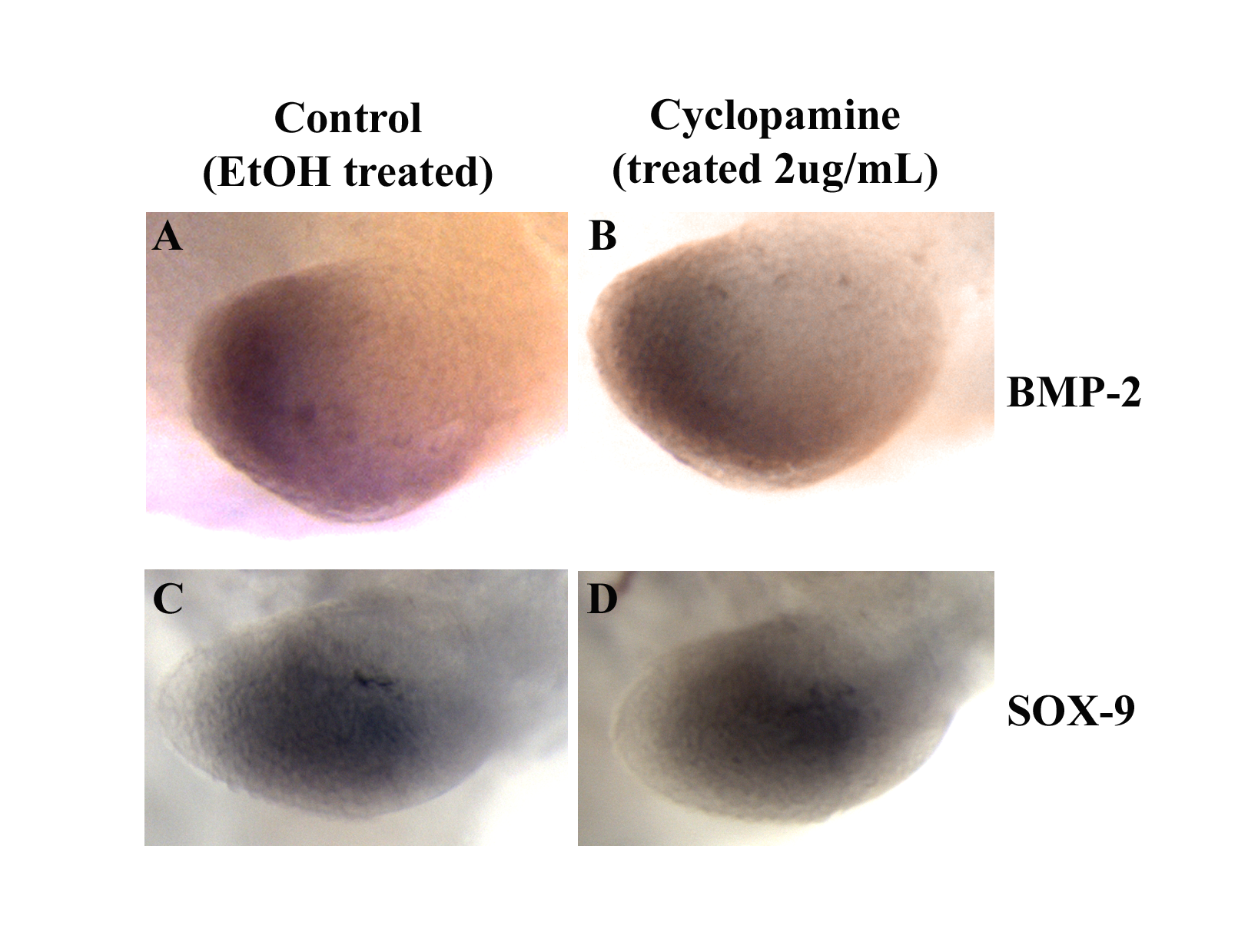

Supplement: Additional file 2 — Figure S2: Expression of BMP-2 and SOX-9 at stage 45 of limb development. Expression of BMP-2 and SOX-9 at stage 45 of limb development in control and cyclopamine treated (2 μg/mL) axolotls. Shh has been shown to be expressed at this stage of axolotl limb development. Our results indicate that the expression of BMP-2 and SOX-9 during limb development is not dependent on Shh signaling as they are not affected by cyclopamine treatment. Panels A and B show the expression of BMP-2 in control (A) and cyclopamine treated (B) animals. Panels C and D show the expression of SOX-9 in control (C) and cyclopamine treated (D) animals. [file 1471-213X-10-15-S2.TIFF]

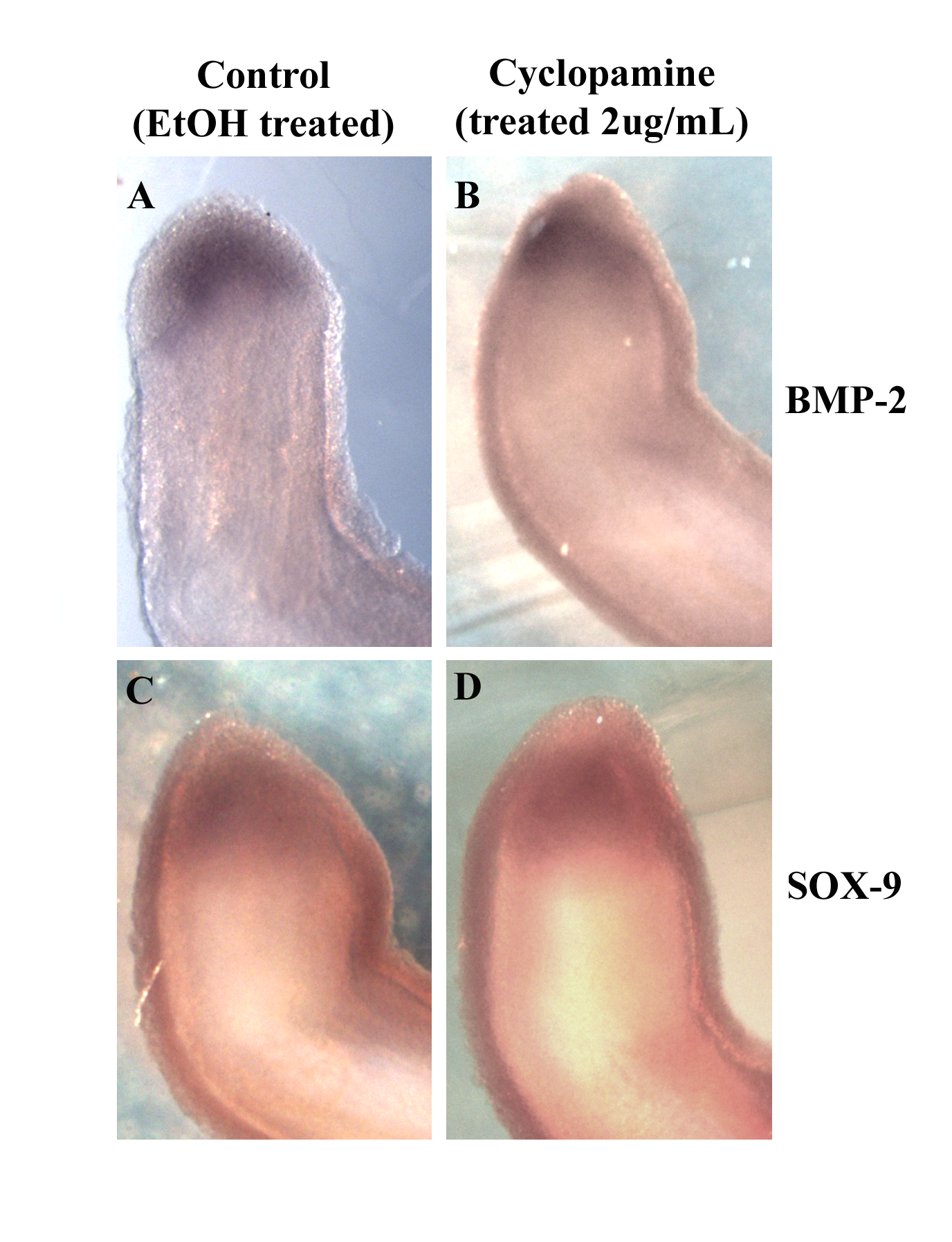

Supplement: Additional file 3 — Figure S3: Expression of BMP-2 and SOX-9 at MB with cyclopamine. Expression of BMP-2 and SOX-9 at MB stage of limb regeneration in control and cyclopamine treated (2 μg/mL) axolotls. Shh has been shown to be expressed at this stage of axolotl limb regeneration. Our results indicate that the expression of BMP-2 during limb regeneration is not dependent on Shh signaling as it is not affected by cyclopamine treatment. Panels A and B show the expression of BMP-2 in control (A) and cyclopamine treated (B) animals. Panels C and D show the expression of SOX-9 in control (C) and cyclopamine treated (D) animals. [file 1471-213X-10-15-S3.TIFF]

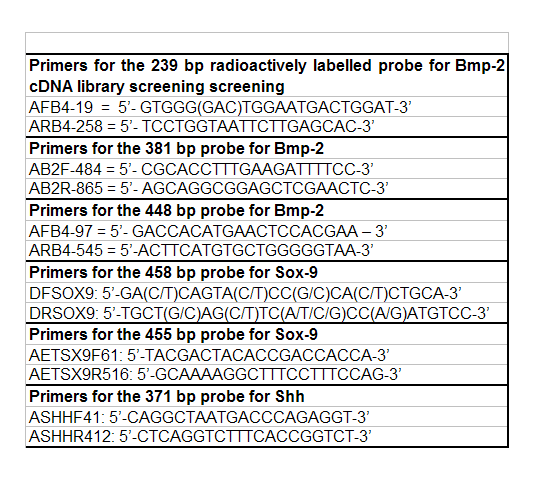

Supplement: Additional file 4 — Figure S4: Primers. Primers used for PCR amplification of the different probes used for whole mount in situ hybridization and cDNA library screening. [file 1471-213X-10-15-S4.TIFF]

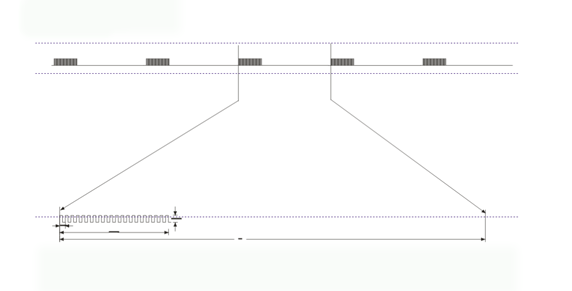

Supplement: Additional file 5 — Figure S5: In vivo electroporation. Schematic representation of the electric pulses used for electroporating the plasmids in regenerating tissues. 5 trains of pulses were applied for the in vivo electroporation of expression constructs. For each electroporation, a train of 20 square waves (10 V peak to peak) in 100 ms (200 Hz) is applied every second (1 Hz) over five seconds (5 trains in total). [file 1471-213X-10-15-S5.TIFF]
